# 1658. Rational use strategy for clarithromycin in a pediatric hospital: is it used more than is required?

**DOI:** 10.1093/ofid/ofad500.1491

**Published:** 2023-11-27

**Authors:** Juan Pablo Londono-Ruiz, Julian Enrique Avila Lombo, Maria Alejandra Medina Losada, Ivan Felipe Gutierrez-Tobar, Andrea Esperanza Rodriguez-hernandez

**Affiliations:** Clinica Infantil Colsubsidio, Staphylored Colombia, Bogota, Distrito Capital de Bogota, Colombia; Universidad del Rosario, Bogota, Distrito Capital de Bogota, Colombia; Universidad del Rosario, Bogota, Distrito Capital de Bogota, Colombia; Clínica Infantil Santa Maria del Lago y Clinica Infantil Colsubsidio, Bogota, Distrito Capital de Bogota, Colombia; Clinica Infantil Colsubsidio, Bogota, Distrito Capital de Bogota, Colombia

## Abstract

**Background:**

Clarithromycin is a macrolide used to treat microorganisms that cause atypical pneumonia or whooping cough in pediatrics. Studies have shown that its use in typical pneumonia does not change clinical outcomes. The implementation of an Antimicrobial Stewardship (ASP) strategy directed at this antibiotic and its rational use allows for reductions in its unnecessary consumption. The aim of this work is to show the impact of an antibiotic control intervention on the use of clarithromycin in a pediatric hospital.

**Methods:**

A before-and-after study was carried out in between 2018 and 2022. In 2020 begins the intervention period during which several educational measures aimed at the clarithromycin formulation were implemented, including a virtual training space, a graphic piece for knowledge of the tools, and the implementation of an audit and feedback system for all clarithromycin formulations. Special emphasis was placed on the differential diagnosis of atypical pneumonia caused by viruses and the promotion of the oral route for this antibiotic. Antibiotic consumption was measured using Days of Therapy (DOT) and adherence to local prescription guidelines. R (version 4.2.2) was used for statistical analysis.

**Results:**

Two years prior to the intervention (2018 and 2019) were analyzed, 2020 was taken as the washout year, and two years after the intervention (2021 and 2022) were analyzed. The average DOT in the pre-intervention period was 10 DOT x 1,000 hospital discharges, compared to the post-intervention period where it was 0.8 DOT x 1,000 hospital discharges (p value < 0.001). In the pre-intervention years, a peak in formulation related to the respiratory peak was observed (between March and May) (Figure 1). Adherence to local prescription guidelines increased from 60% to 95% in the post-intervention period (p value < 0.05).

**Figure:**
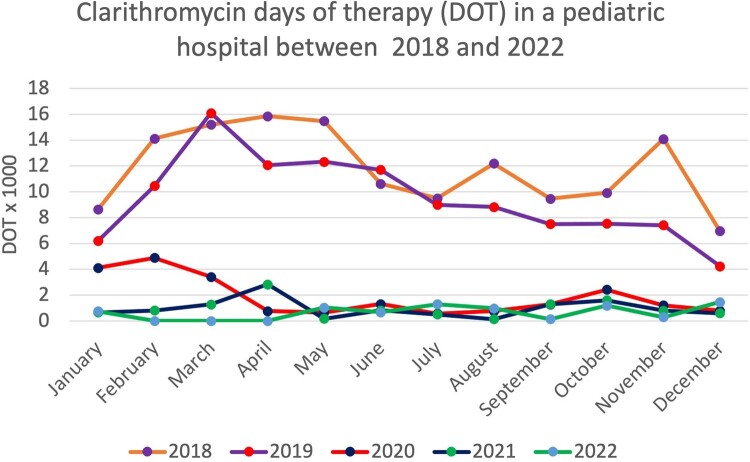

Days of Therapy (DOT) x 1.000 hospital beds between 2018 and 2022. A decrease in the consumption of antibiotics is shown after the implementation of ASP strategy in February 2020

**Conclusion:**

The use of the tools of an antibiotic control program aimed at the use of clarithromycin showed a decrease in the consumption of this antibiotic and increased adherence to the prescription guidelines. It is possible that, on many occasions, the formulation of this was unnecessary.

**Disclosures:**

**All Authors**: No reported disclosures

